# A 36-year-old lady with type three female genital mutilation (Infibulation) – its long-term complications: a case report and literature review

**DOI:** 10.1186/s12905-023-02289-0

**Published:** 2023-05-05

**Authors:** Tafese Dejene Jidha, Abdi Kebede Feyissa

**Affiliations:** 1 College of Medicine and Health Sciences, Dire Dawa University, Dire Dawa, Ethiopia; 2 Department of Pediatrics, Dilchora Referal Hospital, Dire Dawa, Ethiopia

**Keywords:** Female genital mutilation, Childhood, Depression, Infibulation

## Abstract

**Background:**

Female genital mutilation comprises all procedures involving the partial or total removal of female external genitalia or other injury to the female external organs, whether for religious, cultural or other non-therapeutic reasons. The impact of female genital mutilation is diverse, including physical, social and psychological impact. We report a case of a 36-year-old woman with type three female genital mutilation who did not seek medical treatment due to lack of awareness that there was treatment for it, and use this case as an entry point to comprehensively review literature regarding long-term complications associated with female genital mutilation and its impact on women’s quality of life.

**Case presentation:**

We present a case of a 36-year-old single nulligravida lady with type three female genital mutilation who had presented with difficulty with urination since childhood. She had difficulty with menstruation since her menarche, and she had never had sexual intercourse. She never sought treatment, but recently went to hospital after she heard of a young lady who had the same problem in her neighborhood who was treated surgically and got married. On external genitalia examination, there was no clitoris, no labia minora, and labia majora were fused to each other with a healed old scar between them. There was a 0.5 cm by 0.5 cm opening below the fused labia majora near to the anus through which urine was dribbling. De-infibulation was done. Six months after the procedure, she was married and at that moment she was pregnant.

**Conclusion:**

The physical, sexual, obstetrics and psychosocial consequences of female genital mutilation are neglected issues. The improvement of women’s socio-cultural status in combination with planning programs to enhance their information and awareness as well as trying to change the cultural and religious leaders’ viewpoints regarding this procedure is essential to reducing female genital mutilation and its burden on women’s health.

## Background

Female genital mutilation (FGM) comprises all procedures that involve the partial or total removal of external genitalia or other injury to the female genital organs for non-medical reasons [1]. FGM is a violation of girls’ and women’s human rights. More than 200 million girls and women alive today have undergone FGM in 30 countries in Africa, the Middle East and Asia [2]. The reasons why FGM is practiced are a mix of cultural, religious and social factors within the families and communities. They include: psychosexual reasons that aim to limit the sexual desire of women and maintain their virginity, social reasons which include initiation hygiene, rites and aesthetic reasons, myths about the enhancementof fertility, and religious reasons [3]. It is associated with both short-term and long-term complications. Short-term health risks include severe pain, haemorrhage, infection, and in its worst cases, death, while long-term complications entail the formation of cysts,keloids, chronic pelvic infection, sexual dysfunction and obstetric problems [4, 5]. It was thought that in most countries with available data that FGM should end and there has been an overall decline in the prevalence of the practice over the last three decades, but not all countries have made progress and the pace of decline has been uneven.Moreover, despite numerous efforts accomplished globally to enable policies and establish effective strategies for preventing FGM, key knowledge gaps reamin to deliver optimal evidence-based care to improve health outcomes for girls and women affected with FGM. We report a case of 36-year-old woman with type three female genital mutilation who did not seek medical treatment due to lack of awareness that there was treatment for it, and use this case as an entry point to comprehensively review literatures regarding long-term complications associated with FGM and its impact on women’s quality of life.

## Case presentation

A 36-year-old Ethiopian single nulligravida lady was presented to Dilchora Referral Hospital with difficulty of urination since childhood. She had to stay in the toilet for hours for urination. The urine passed dribbling in a very slow stream. She had repeated episodes of pain during urination which was associated with suprapubic lower abdominal pain for which she was given tablets at a nearby health center. She also had the sensation of incomplete bladder emptying following urination. Because of this, she limited fluid intake, especially when she had long-distance travel. She had also difficulty with menstruation since her menarche (i.e., at age 13 years). She had severe cramping lower abdominal pain during her menses, and her menses stayed for seven to eight days. She had a history of genital cutting 30 years ago at five years of age. She never had sexual intercourse. She was proposed many times to different men but refused to marry because of fear that she could not have sexual intercourse with her husband and could not give birth after marriage. She is living with her younger brother, who is married and has four children. Her mother died when she was nine years old, and her father died ten years ago. She had never told her problem to anyone, and she never sought treatment, but recently went to the hospital after she heard of a young lady who had the same problem in her neighborhood who had been treated surgically and got married.

**Fig. 1:**
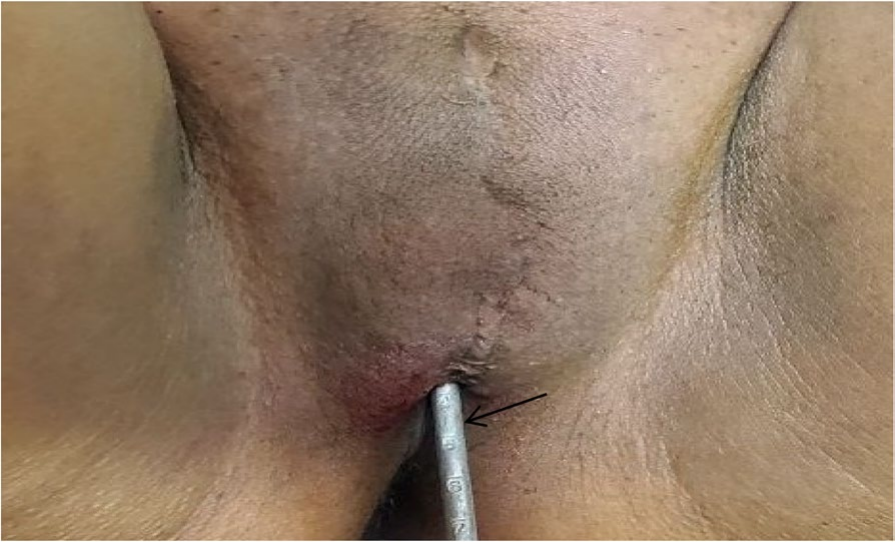
There was no clitoris, no labia minora, and labia majora were fused with a healed old scars between them. There was a 0.5 cm by 0.5 cm opening below the fused labia majora near to the anus that showed with a uterine sound (arrow)

On physical examination, she was depressed, and her vital signs were in normal range. A pertinent finding was detected on genitourinary system: on external genitalia examination, there was no clitoris, no labia minora, and labia majora were fused to with a healed old mild scars between them. There was a 0.5 cm by 0.5 cm opening below the fused labia majora near the anus through which urine was dribbling (Fig. 1).

With the impression of type three FGM (Infibulation), the patient was investigated with complete blood count (all in the normal range), urine analysis (non-revealing), and blood group and Rh (A positive). An abdominopelvic ultrasound examination was also performed. It revealed a normal uterus that was anteverted and measured 67 × 20 × 35 mm. Both ovaries had normal size and appearance. There was a trace of normal fluid in the Douglas pouch. Both kidneys were in their normal anatomic location and had normal size and corticomedullary differentiation.

**Fig. 2:**
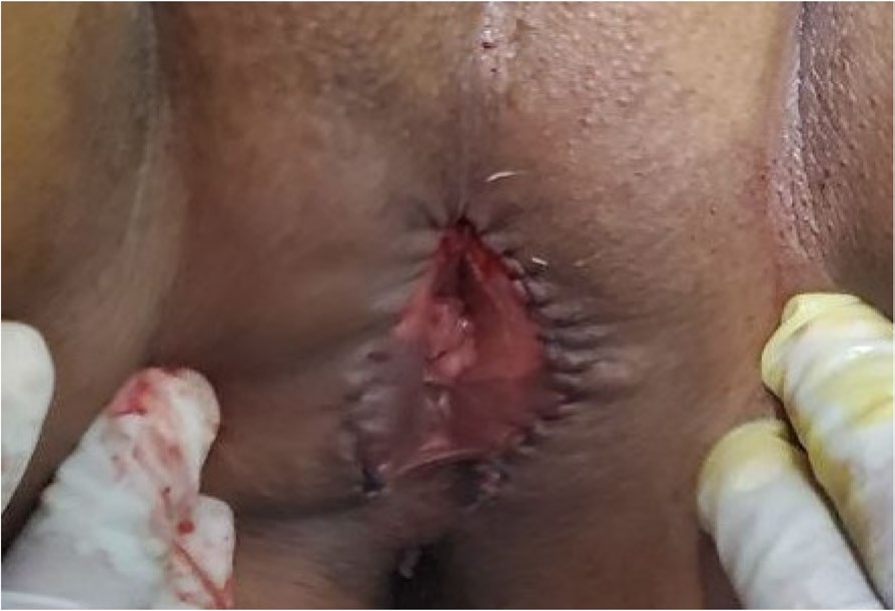
De-infibulation was done and found intact hymen and external urethral opening

Then, verbal informed consent was taken, and under local anesthesia, de-infibulation was done and an intact hymen and external urethral opening were seen (Fig. 2). She was also provided with psychological counseling interventions by a psychiatrist. She was discharged a day after the procedure with diclofenac tablets with an appointment to come after seven days.

Her first follow-up was on her seventh day after the procedure, during this time, she had no difficulty with urination, and on genital examination, the wound had healed. Her second follow-up visit was a month after the procedure, during this time, she was on her seventh day after mense which flowed for four days and was associated with mild abdominal discomfort. Her third follow-up visit was six months after the procedure, during this time, she was married with good wedding adjustment and started intercourse with her husband, and she complained of moderate pain during intercourse, especially with the initial insertion of the penis. She was also pregnant, and her obstetrics ultrasound revealed the first trimester, a single intrauterine pregnancy with a gestational age of six weeks and six days. After the third visit, she missed her follow-up, and nothing was known about her pregnancy and its outcome.

## Discussion and conclusion

This case report describes a case of a 36-year-old single nulligravida lady with type three female genital mutilation (infibulation) who was depressed, socially isolated, presented with difficulty with urination since childhood, and had difficulty with menstruation since her menarche, for whom de-infibulation was done, and provided with psychological and counseling interventions, after which she got married, and became pregnant.

Females’ genital mutilation (FGM) is one of the harmful traditional practices affecting the health of children and women. It remains a serious problem for a large proportion of children and women in most sub-Saharan African countries including Ethiopia. In addition to acute complications, it also has a long-term physiological, psychological, and sexual effects on children and women. Management of patients with infibulation requires not only de-infibulation, but also psychological and counseling interventions.

Immediate complications of female genital mutilations are bleeding, immediate pain, swelling, shock and infection, including wound infection, sepsis, gangrene and tetanus [6, 7]. It is increasingly recognized that FGM causes complications throughout the life span of women and these complications can be broadly divided into three main areas; uro-gynecological, obstetric and psychological (including sexual function) complications.

Long-term uro-gynecological concerns that have been linked to FGM include infection, scarring and keloid, urinary symptoms, menstrual difficulties and infertility [7, 8].

FGM, especially the more severe forms of FGM (infibulation) has been correlated with the higher frequency of infections including recurrent urinary tract infections, genitourinary tract infections, and chronic genital abscesses [9, 10]. The etiology for recurrent genitourinary tract infections is incomplete bladder emptying and bladder outlet obstruction [7]. In our case, she had repeated episodes of pain during urination which was associated with suprapubic lower abdominal pain for which she was repeatedly given tablets at a nearby health center.

Genital scarring is common but can be very variable due to the extent of tissue removed and the presence of immediate complications such as wound infection. The keloid may form over the scar sites and may disfigure the vulva and can be a source of anxiety, embarrassment and fear of surgical treatment to women who had FGM [11]. Inclusion cysts over the clitoral area may form and can obstruct the vagina and urethra causing difficulty in urination and sexual discomfort [12]. The cysts can be very large and require surgical excision. In our case, labia majora were fused with a healed old mild scars between them.

Damage to the urethra during FGM may lead to urogenital fistula and urethral strictures [7, 8]. The risk factors for post-procedure urogenital fistula and urethral strictures are lack of anatomical knowledge by local practitioners, use of non-sterilized crude instruments, traumatic sexual intercourse, recurrent urogenital infection, obstructed labor due to vaginal stenosis, and poor assistance during vaginal delivery [7, 8]. The most common complaints in these cases are urinary incontinence and recurrent urinary tract infection. Poor urinary flow, incomplete bladder emptying, and urinary retention have also been reported in women following FGM and, are thought to be due to post-procedure genital swelling, painful and traumatic experience, and obstruction of the urethral by scar tissue sealing the vagina [5, 7, 13]. There was also a case of total urinary incontinence that was due to urethral intercourse with a stenosed vagina [14]. It was seen that the urethral orifice was so enlarged that the insertion of two fingers was possible. In our case, the patient presented with difficulty with urination since childhood. The urine passed dribbling in a very slow stream. She also had the sensation of incomplete bladder emptying following urination. Because of this, she limited fluid intake, especially when she had long-distance travel. On genital examination, the urethral and vaginal openings were sealed with fused labia majora. After de-infibulation, it was evaluated and had no urogenital fistula, and the patient had improved urinary symptoms.

Patients with severe forms of FGMs (type three) may also present with painful and prolonged periods. It is possible that a very narrow vaginal opening might slow menstrual flow and prolong the duration of bleeding, and rarely develop haematocolpos [7]. Most of the time, these symptoms are relieved by de-infibulation. In our case, the patient had difficulty with menstruation since her menarche. She had severe cramping lower abdominal pain during her menses, and her menses stayed for seven to eight days. During subsequent follow-ups after the procedure, her period flowed for four to five days and was associated with mild abdominal discomfort.

It has been suggested that severe forms of FGM lead to infertility [7, 15]. Difficult or painful intercourse because of the infibulated vagina and ascending pelvic infection with recurrent genitourinary infections in patients with FGM have been suggested as possible mechanism [7, 15, 16]. With multiple coital attempts over several months and using ample lubricants, the scar may stretch, but coitus is still painful, the obstacle that prevents them from getting pregnant. Once pregnant, infibulated women face another challenge during labor and delivery. Women with the severe forms of FGM are at increased risk of prolonged or difficult labor, perineal trauma, operative delivery, and postpartum hemorrhage [16–18]. In addition, there is an increased risk of stillbirth, neonatal resuscitation, and early neonatal death [7]. In our case, the patient never had sexual intercourse before the procedure. She was proposed to many times to different men but refused to marry because of fear that she had no vagina and she could not have sexual intercourse with her husband and could not give birth after marriage. Six months after the procedure, when she came for her third follow-up visit, she was married and started intercourse with her husband, and she complained of moderate pain during intercourse, especially with the initial insertion of the penis. she was also pregnant, and her obstetrics ultrasound revealed the first trimester, a single intrauterine pregnancy with a gestational age of six weeks and six days. After the third visit, she missed her follow-up, and nothing was known about her pregnancy and its outcome.

Studies have shown that women with FGM may be more likely to experience psychological disturbances including posttraumatic anxiety, somatization disorders, phobia, and low self-esteem. Furthermore, they may experience various emotional difficulties including loss of trust between mother-daughter, anger, a feeling of fear, and helplessness [7, 15, 18]. Therefore, preoperative evaluation and counseling for de-infibulation, and psychological support along with surgical interventions are needed to help women manage the physiological and psychological challenges following surgery. In our case, the patient was depressed and socially isolated and she had never told her problem to anyone, and she never sought treatment. After the procedure, during the subsequent follow-up visit, she was provided with counseling and psychological support by a psychiatrist.

Concerning sexual consequences, women with FGM are more likely to experience pain during intercourse, less likely to experience sexual desire, and experience less sexual satisfaction [15]. In our case, six months after the procedure, she started intercourse with her husband, and she complained of moderate pain during intercourse, especially with the initial insertion of the penis.

This case study, using the case as an entry point, provides valuable information about long-term complications associated with FGM and its impact on women’s quality of life, but it is not without limitations. The limitation of this case study was that patient was not followed till delivery because she missed her follow-up after she got pregnant. Therefore it wasn’t easy to comment on her obstetric outcomes.

The physical, sexual, obstetrics and psychosocial consequences of FGM are neglected issues. Improving women’s socio-cultural status, combined with planning programs to enhance their information and awareness, and trying to change the cultural and religious leaders’ viewpoints regarding this procedure, is essential to reduce FGM and its burden on women’s health.

## Patient’s perspective

I was five years old when it happened to me. I have suffered a lot from the day I was cut. For me, to mutilate a girl is to make her suffer for the rest of her life. I think FGC is ridiculous. I think about what happened to me every day. I wish girls would not suffer from this absurd practice. Thanks to doctors who helped me, I am urinating without difficulty, and my menses comes monthly and flows without problem; I am also married and got pregnant and expecting a baby. But I still have scars in my mind from the suffering I have passed through over the past 30 years.

I hope girls will not suffer from this traditional practice anymore, and all stakeholders, including religious leaders and community figures, should work together to prevent this harmful traditional practice. I am happy that people worldwide are learning from my case, and I do not mind my condition being discussed. I do not want anyone to go through what I had to.

## Data Availability

Data sharing is not applicable to this article as no datasets were generated or analyzedduring the current study.

## Abbreviations

FGM: Female genital mutilation
WHO: World Health Organization
HIV: Human Immunedeficiency Virus
